# Humanitarian and primary healthcare needs of refugee women and children in Afghanistan

**DOI:** 10.1186/s12916-017-0961-y

**Published:** 2017-12-11

**Authors:** Ariel Higgins-Steele, David Lai, Paata Chikvaidze, Khaksar Yousufi, Zelaikha Anwari, Richard Peeperkorn, Karen Edmond

**Affiliations:** 1 UNICEF Afghanistan Country Office, Kabul, Afghanistan; 2 World Health Organization Afghanistan, Kabul, Afghanistan; 3 Afghanistan Ministry of Public Health, Kabul, Afghanistan; 4 UNICEF Afghanistan, United Nations Office Complex in Afghanistan (UNOCA), Jalalabad Road, Kabul, Afghanistan

**Keywords:** Refugees, Returnees, Afghanistan, Women, Children

## Abstract

This Commentary describes the situation and healthcare needs of Afghans returning to their country of origin. With more than 600,000 Afghans returned from Pakistan and approximately 450,000 Afghans returned from Iran in 2016, the movement of people, which has been continuing in 2017, presents additional burden on the weak health system and confounds new health vulnerabilities especially for women and children. Stewardship and response is required at all levels: the central Ministry of Public Health, Provincial Health Departments and community leaders all have important roles, while continued support from development partners and technical experts is needed to assist the health sector to address the emergency and primary healthcare needs of returnee and internally displaced women, children and families.

## Commentary

**Fig. 1:**
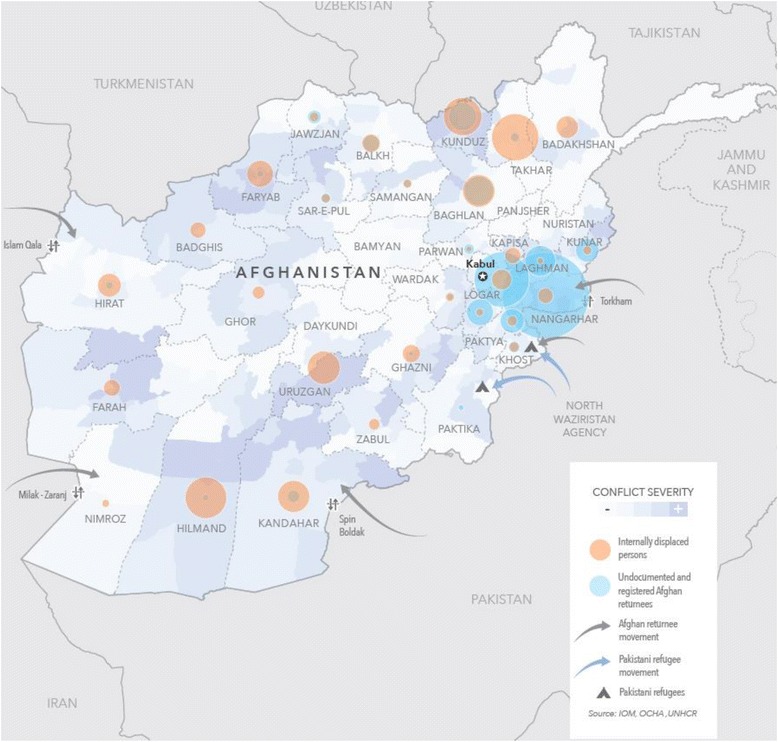
Population movement in Afghanistan (source: IOM, OCHA, UNHCR 2017)

While much attention understandably focuses on refugees and migrants entering Europe, many Afghan children, women and their families are forcibly or voluntarily returning to Afghanistan from Europe and neighbouring countries, including Pakistan and Iran. Commonly called ‘returnees’, these mothers and children are among the most vulnerable of all refugees, requiring support and investment across sectors, particularly for health. In 2016, more than 600,000 Afghans returned from Pakistan, whereas approximately 450,000 Afghans returned from Iran (Fig. 1) [1, 2]. Further, in addition to the returnees, there is a large group of internally displaced persons (IDPs) in Afghanistan. All regions in Afghanistan have been affected by the conflict and therefore 1500 people per day, on average, are forced to leave their homes to escape violence. In the first quarter of 2017, there were approximately 68,000 newly IDPs in the country [3, 4]. With no foreseen improvements in security, 450,000 new IDPs and approximately 1 million returnees from Pakistan alone are expected in 2017 [1].

Due to the ongoing conflict in Afghanistan, both returnees and IDPs require routine primary healthcare as well as emergency health services. Recent estimates suggest that more than 7 million people have no or limited access to essential health services due to insufficient coverage by the public health sector and direct interruption due to conflict and insecurity [1, 5]. Immediate health needs support for refugee and IDP children, women and their families in Afghanistan includes vaccines (including poliovirus and measles vaccine), management of malnutrition, antibiotic treatment of infections, screening for tuberculosis, and prevention and care of sexually transmitted infections and HIV infection. Additionally, practical supplies, such as shelter, food, clothing, cash transfers, hygiene kits, insecticide treated nets and cash transfers for food and basic household items, are also required. Pregnant women (approximately 4.2% of the general population) require antenatal care, clean delivery kits and skilled delivery care. It is estimated that 10–15% of deliveries in Afghanistan will face complications and will require surgical intervention and safe blood transfusion. Skilled postnatal care for mothers and their newborns, as well as supplies and counselling for emergency and postpartum family planning are also needed.

Coordination of the essential emergency health services in Afghanistan is being provided by the Health Cluster, led by the Ministry of Public Health and the World Health Organization with support from various partners. Encashment centres are one point of contact where support is delivered, as well as through zero points (at national borders), fixed health centres and mobile health teams. Yet, there are many challenges in providing an appropriate health sector response to women and children in Afghanistan. Afghanistan has a severely under-resourced public health system; while there have been improvements, the geographic distribution of health centres in the country is uneven and many families and pregnant women live more than 2 hours walking distance from a health facility [6]. For example, only approximately 50% of all pregnant women in Afghanistan deliver their babies in a health facility with a skilled attendant in contrast to the 70–80% rates reported in many low-income countries [6, 7]. Further, much of Afghanistan is mountainous and conflict in many areas of the country is increasing, additionally hampering access of vulnerable children and women to life-saving health services. Health facilities are understaffed, with few female staff, including night duty workers, vaccinators and nurses to provide maternal and newborn care. Financial pressures and conflict are causing returnee and internally displaced families to be constantly ‘on the move’ across Afghanistan. Children and their mothers are likely to miss important follow-up medical appointments and treatment. In addition, unlike other refugees, families who arrive in Afghanistan may experience a more violent, unstable and stressful environment than in their country of departure. This situation contributes to the financial, physical and emotional ‘shocks’ that Afghan refugee families face.

**Fig. 2:**
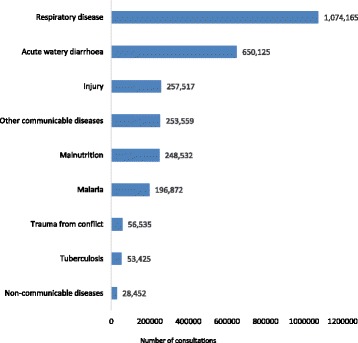
Primary health care consultations for children under five years of age in Afghanistan, July 2016 to June 2017 (source: Afghanistan Ministry of Public Health, Health Management Information System)

Indeed, the large numbers of families arriving are now pushing Afghanistan’s health system to its limits, especially in the host communities (Fig. 2). An assessment undertaken in April 2017 in Nangarhar, a province bordering Pakistan, found that the caseload of in-patient departments in the regional hospital had increased by 27% and out-patient consultations had increased by 41% compared to the same period in 2016 [8]. In the same assessment, the district hospital bed occupancy rate was reported to be beyond the maximum range of 100%. In particular, the paediatric and maternity ward average bed occupancy rates from January to March 2017 were 145% and 115% per month, respectively (Personal communication with Nangarhar Province Department of Health, May 2017).

## Conclusion

Stewardship and response is required at all levels; the Ministry of Public Health, Provincial Health Departments and community leaders all have important roles. Continued support from development partners and technical experts is needed to assist the health sector to address the emergency and primary healthcare needs of returnee and internally displaced women, children and families. Mental healthcare, emotional support, and child and family protection are additional but critical needs in a country already struggling to provide basic emergency and primary healthcare for its most vulnerable populations.
